# Sunburn-induced bark damage facilitates *Eutypella decipiens* infection of *Carpinus betulus* in Serbian urban landscapes

**DOI:** 10.3389/fpls.2026.1828539

**Published:** 2026-05-11

**Authors:** Dušan Jovanović, Slobodan Milanović, Thomas Jung, Josef Janoušek, Miloš Trifković, Zoltán Á. Nagy, Marília Horta Jung, Ivan Milenković

**Affiliations:** 1 Faculty of Forestry, University of Belgrade, Belgrade, Serbia; 2 Department of Forest Protection and Wildlife Management, Mendel University in Brno, Brno, Czechia; 3 Phytophthora Research and Consultancy, Nußdorf, Germany

**Keywords:** abiotic stress, *Anthostoma* spp., bark cankers, hornbeam, pathogenicity, solar radiation

## Abstract

European hornbeam (*Carpinus betulus*) is a significant component of urban green spaces in Serbia. Recently, a decline characterized by crown dieback and distinct spindle-shaped bark cankers has been observed in planted alley trees. This study aimed to identify the causal agent of these symptoms and investigate the role of environmental stressors in disease development. Field surveys were conducted in central Serbia, assessing the orientation and morphology of cankers. Following isolation and molecular identification, *Eutypella decipiens* (previously known as *Anthostoma decipiens*) was identified as the causal agent of bark cankers and the decline. Pathogenicity was tested using two-year-old *C. betulus* saplings inoculated with *E. decipiens* and, for comparison, *Cryphonectria carpinicola*, another bark pathogen on this host. Inoculation sites were monitored weekly for six weeks in a controlled glasshouse environment. Field observations revealed that cankers were exclusively located on the south and southwest sides of trunks, suggesting a correlation with sunburn damage. In pathogenicity trials, *E. decipiens* successfully colonized host tissues, with over 50% of plants exhibiting dieback symptoms within six weeks. The recorded necroses were on average 10.2 times longer and 22.9 times larger in area than the control. Necroses in the *C. carpinicola* treatment were smaller being 2.9 times longer and 6.1 times larger in area compared to the control. Our findings demonstrate that sunburn-induced bark damage may facilitate *E. decipiens* infection highlighting the enhanced vulnerability of urban *C. betulus* populations to synergistic climate-driven stressors. This study provides the first evidence of an abiotic-mediated infection pathway for this pathogen and represents its initial record within urban landscapes, extending its known range beyond the natural forests of Serbia. Implications of these findings and general recommendations to reduce the damage are discussed.

## 1 Introduction

The European hornbeam (*Carpinus betulus* L.) is an ecologically important and widespread species across temperate Europe (Finnie et al., 2007; Sikkema et al., 2016). According to Cvjetićanin and Perović (2016), *C. betulus* is naturally distributed in central, south and partly in western Europe, southeast Asia and the Caucasian area where it occurs until Iran (Abdi et al., 2009). This species is showing high adaptivity to various site conditions (Bobinac, 2004) and has a wide ecological amplitude. Naturally, it grows in mixed stands usually accompanying noble hardwoods and, according to Banković et al. (2009), the most common species in mixture with hornbeam in Serbia are European beech (*Fagus sylvatica* L.), Turkey oak (*Quercus cerris* L.), sessile oak (*Q. petraea* (Matt.) Liebl.), Hungarian oak (*Q. frainetto* Ten.), manna ash (*Fraxinus ornus* L.), field maple (*Acer campestre* L.) or linden species (*Tilia* spp.). Also, in lowland floodplain forests *C. betulus* appears as a hygrophilic companion of English oak (*Q. robur* L.). Besides natural ecosystems, European hornbeam is widely grown in ornamental nurseries and its numerous varieties and forms are frequently planted in urban areas.

Since the beginning of the 21st century, a decline in *C. betulus* has been recorded in various natural and urban ecosystems across Europe, primarily in Italy and Central Europe, as summarized by Brühwiler (2021) and Cornejo et al. (2021). Several recent reports have also indicated further incidences of decline symptoms in Europe (Muser and Burgdorf, 2022; Papp et al., 2024; Milenković et al., 2024; Nuskern et al., 2025; Remmele et al., 2025). This suggests that although it is considered tolerant to pathogens, widely distributed in natural ecosystems, and frequently used in urban areas for ornamental purposes, *C. betulus* is actually highly susceptible to parasitic and saprotrophic organisms, particularly due to various stress factors. For instance, in a comprehensive study of fungal diversity on *C. betulus* in natural ecosystems in Serbia, 55 different parasitic and saprotrophic fungi were recorded (Karadžić, 2011), including five species on leaves, 22 species on bark, and 28 wood-decay and rot fungi.

Among the fungal pathogens associated with *C. betulus*, *Eutypella decipiens* (Nitschke) Dissan., Hyde & Liu, previously known as *Anthostoma decipiens* (DC.) Nitschke, has been identified as the causal agent of bark cankers and tree decline across Europe (Rocchi et al., 2010; Saracchi et al., 2015; Muser and Burgdorf, 2022; Remmele et al., 2025) and in Iran (Mirabolfathy et al., 2018). Another bark pathogen causing similar symptoms, *Cryphonectria carpinicola* Rigling, Cech, Cornejo and Beenken, previously known as *Endothiella* sp. (Saracchi et al., 2015), has been recently recorded in several European countries (Cornejo et al., 2021, 2023a, 2023b; Papp et al., 2024; Milenković et al., 2024; Nuskern et al., 2025). In addition, bacterial pathogens (e.g., those locally associated with acute oak decline), root rot and leaf pathogens, such as powdery mildews and *Colletotrichum* spp., contribute to the overall disease pressure on *C. betulus* (Braun et al., 2006; Moradi-Amirabad et al., 2019; Chinan and Mânzu, 2021; Mao et al., 2021; Qiao et al., 2023).

While monitoring tree health across various urban areas in Serbia, specimens of *C. betulus* were observed exhibiting crown decline, branch dieback and spindle-shaped bark cankers of relatively uniform size and position. These observations raise important questions about the causal agents and the role of environmental stressors, such as sun exposure and heat stress, in facilitating infection. Therefore, the aims of this study were to: i) isolate and identify the causal agents of these decline symptoms, with particular attention to the potential contribution of abiotic stressors such as sunburn; ii) test the aggressiveness of the isolated organisms on *C. betulus* saplings.

## 2 Materials and methods

### 2.1 Study area and disease symptoms

**Figure 1:**
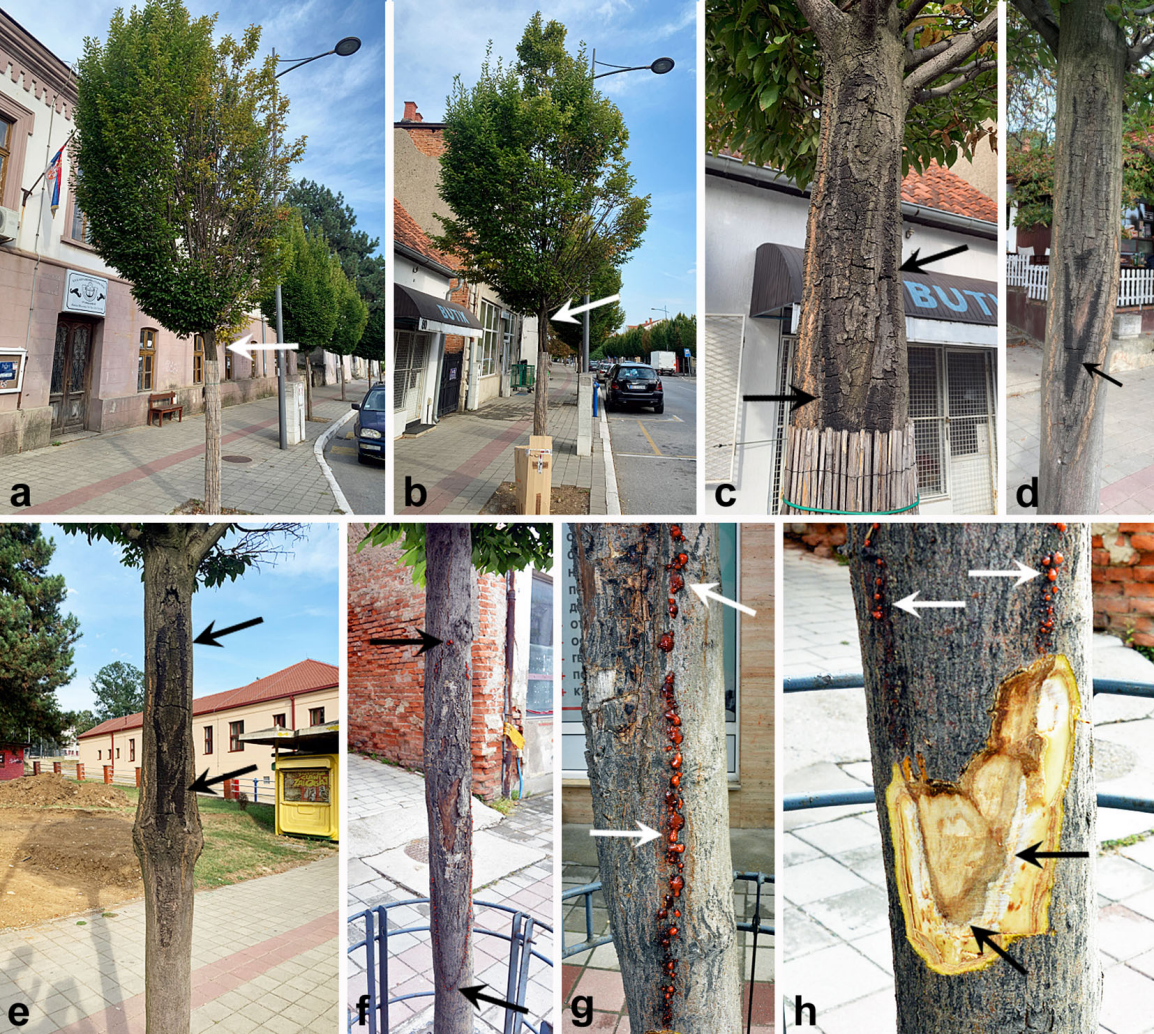
Symptoms of *Carpinus betulus* trees infected with *Eutypella decipiens* in an urban area in Serbia: **(A, B)** increased crown transparency, wilting and beginning dieback (arrows indicate the upper margins of the cankers); **(C–E)** elongated, elliptic-to-spindle-shaped cankers on *C. betulus* stems, showing fruiting bodies within stromata; **(F, G)** cankers with numerous reddish to red-orange cirrhose tendrils at the margins (arrows); **(H)** reddish to red-orange cirrhose tendrils at the canker margins (white arrows) and necrotic cambial tissue beneath the bark with visible active margins of lesion development (black arrows).

**Figure 2:**
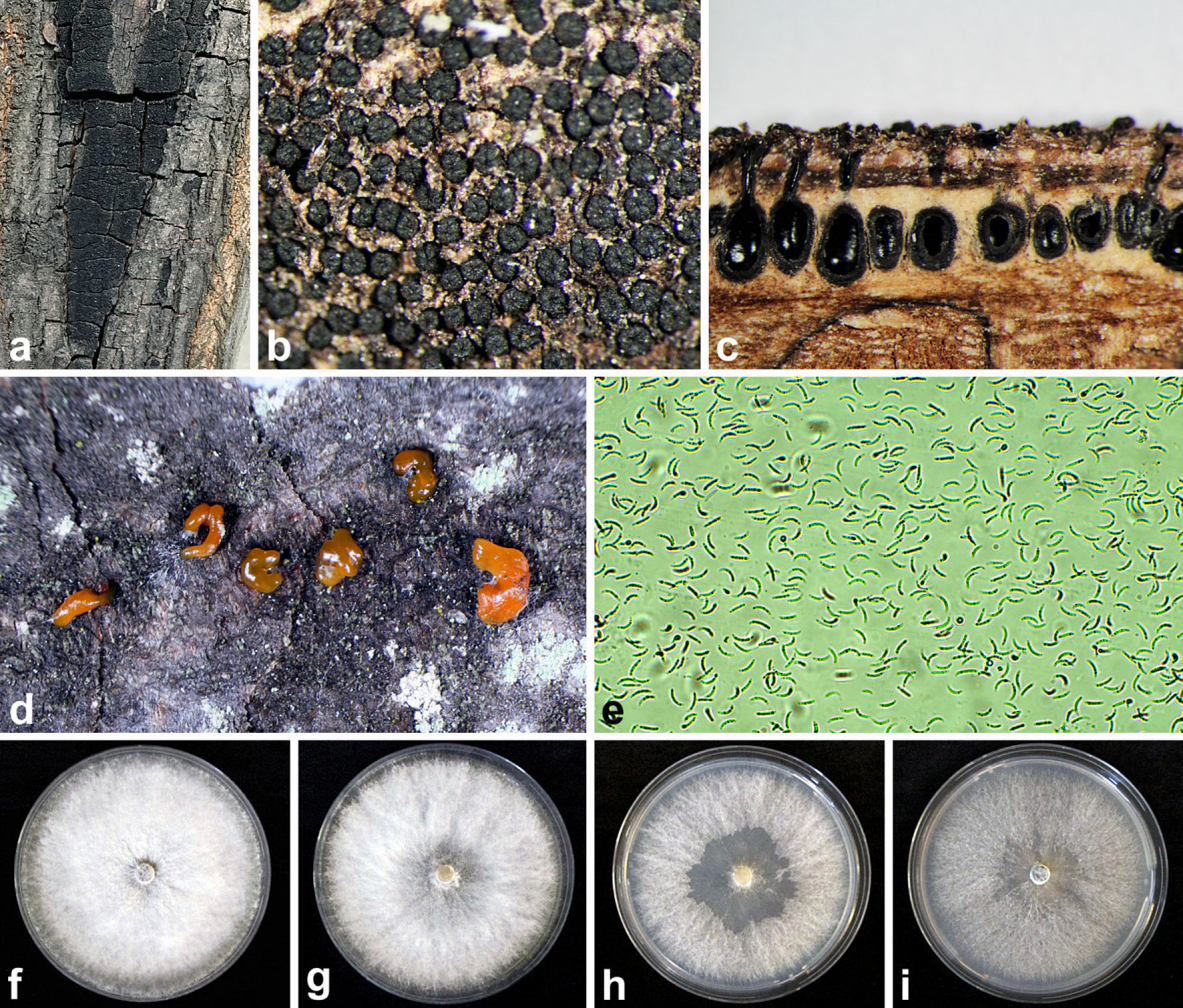
Morphology of the *E. decipiens* and colony growth patterns: **(A)** Perithecial stromata on dead bark of *C. betulus*; **(B)** close-up of perithecial furrowed apices; **(C)** transverse section of stroma and host tissue showing immersed perithecia; **(D)** close-up view of cirrhi (tendrils) under a stereomicroscope; **(E)** numerous allantoid, unicellular, and hyaline conidia; **(F–I)** colony morphology on PDA, MEA, V8A, and CA, respectively, after 14 days of incubation at 20 °C in the dark.

The study was conducted in the urban area of the Aranđelovac municipality, specifically focusing on the *Carpinus betulus* ‘Fastigiata’ alley trees on Knjaz Miloš Street in May 2021. This street is oriented in a northwest-southeast direction (**Supplementary Figure 1**), so that the side of the street oriented to south-southwest is exposed to increased sun radiation compared to the opposite side which is protected by residential buildings. Some of the trees displayed dieback symptoms of branches and crowns (**Figures 1A, B**) and 50 trees in this alley showed regular elongated ellipsoid to spindle-shaped and ca. 1 m long lesions on the stems at approximately 1.5 m from the stem base (**Figures 1C–F**). Basically, all the trees (100%) at the sun-exposed side of the street showed cankers and lesions that were regularly oriented to the south-southwest. At the edges of these cankers, numerous reddish to red-orange tendrils were recorded (**Figures 1F–H**), whereas active necrotic zones were recorded below the necrotic bark (**Figure 1H**). Also, on the dead necrotic bark numerous stromata with fruiting bodies were recorded (**Figures 1C–E**; **Figures 2A–C**). Some trees showed smaller cankers whereas in some cases wood decay fungi were recorded on the affected tissues (**Supplementary Figure 2**). The average annual precipitation for the period 1990–2020 in the area of Aranđelovac was 680.8 mm, while the average temperature was 11.7 °C (https://atlas-klime.eko.gov.rs). Global horizontal irradiance (GHI) as the amount of terrestrial irradiance falling on a surface horizontal to the surface of the earth was 1348.7 kWh/m2 (https://globalsolaratlas.info/map).

### 2.2 Sampling, isolation and morphological identification

Twenty out of 50 trees (40%) that were affected with the disease symptoms in the Knjaz Miloš street in Aranđelovac were randomly selected and sampled in May 2021. On each tree, a single representative stem canker was selected for sampling. Necrotic bark samples containing fruiting bodies were collected from both the upper and lower active margins of stem cankers (**Figure 1H**) using a sterilised knife. This resulted in a total of 40 samples (two per tree), which were then transported to the laboratory for analyses. Stromata and conidiomata that were present on the bark surface were examined using a Olympus SZX7 stereomicroscope (Olympus Europa, Hamburg, Germany) equipped with an EOS 3000D digital camera (Canon, Japan). Conidia were measured and recorded using Ceti Magnum-T trinocular light microscope (Medline Scientific Ltd., United Kingdom) and recorded using a Si3000 digital camera (Fisher Scientific, United Kingdom), equipped with XliCap^®^ (Xl Imaging Ltd., United Kingdom) software.

The collected bark samples were cut and the surface was sterilized by immersion into 70% ethanol for 1 min. After drying on sterilized filter paper in the laminar flow, tissue pieces ca. 5 × 5 × 2 mm in size were taken using a sterilized scalpel from the transition zones between necrotic and healthy tissue, additionally surface sterilized by passing through the opened flame and plated onto 2% MEA medium (20 g/L of Malt Extract (HiMedia, India) and 20 g/L of Agar (Torlak, Serbia)). A total of 10–15 small pieces per sampled canker were plated and incubated at 20 °C in the dark. Plated samples were observed daily under the stereomicroscope and when first hyphae appeared they were immediately transferred onto fresh MEA using a sterilized needle and kept at 20 °C in the dark for further analysis. All isolates were stored on 2% MEA at 4 °C in the Faculty of Forestry culture collection at the University of Belgrade (UBFF).

To determine colony morphology, two selected isolates (UBFF161 and UBFF234) from hornbeam were developed on MEA. Using a sterilized, 7 mm diameter metal cork borer, isolates were transferred onto four different media including Potato Dextrose Agar (PDA; HiMedia, India), MEA, Vegetable juice agar (V8-A) and Carrot agar (CA) (prepared with 100 ml/L of V8 and CA juice, respectively (Biotta^®^, Swiss), and 20 g/L of Agar (Torlak, Serbia)). Petri dishes were incubated at 20 °C in the dark and colony morphologies were recorded after 14 days.

Growth rates and cardinal temperatures were determined for the two selected isolates on MEA and PDA, incubated at nine different temperatures, including 10, 15, 20, 25, 27.5, 30, 32.5, 35 and 37.5 °C. Three replicates per isolate and temperature were made. After initial development for 24 h at room temperature (20−22 °C), the isolates were transferred to the incubators with the respective temperatures for another 24 h. At the bottom of each Petri dish, two perpendicular lines were made with a 0.5 mm marker pen and colony development was denoted with steel needle after every additional 24 h for five days or until the isolates covered the Petri dishes. Isolates showing no growth at certain temperatures within five days were returned to 20 °C to determine whether these temperatures were lethal. Daily growth rates were calculated using MS Excel (Microsoft, USA).

### 2.3 Molecular identification

To identify the obtained isolates, two pure isolates (UBFF161 and UBFF234) were randomly selected, transferred onto PDA, and incubated at 20 °C in the dark for 10 days. Mycelium was scraped from fresh fungal tissue, and genomic DNA was extracted using the Monarch^®^ Spin gDNA Extraction Kit (New England Biolabs, Ipswich, USA), according to the protocol described by Jung et al. (2022). The *β*-tub (Glass and Donaldson, 1995), *cmdA* (Duong et al., 2012), *tef1α* (O’Donnell et al., 1998; Carbone and Kohn, 1999), and ITS (White et al., 1990) regions were amplified using the primers and conditions listed in **Supplementary Table 1**. PCR protocol was as follows: 20 µl volume containing 10.4 µl H_2_O, 4 µl Q5 Reaction Buffer (5X), 1 µl of each primer (10 μM), 0.4 µl deoxynucleotide (dNTP) mixture (Meridian Bioscience, Memphis, USA) (2.5 mM each), 0.2 µl of Q5 High-Fidelity DNA Polymerase (2 U/μl) (New England Biolabs, Ipswich, USA), and 3 µl of gDNA. Initial denaturation was for 30 s at 98 °C; 35 cycles consisting of 5 s at 98 °C, 20 s at specific annealing temperature for each primer set, optimised length of extension at 72 °C; 2 min at 72 °C for final extension. PCR amplifications were performed on the LC480 II (Roche, Switzerland). After electrophoresis, the amplicons were purified and sequenced in both directions by Eurofins Genomics GmbH (Cologne and Ebersberg, Germany) using the amplification primers. Electropherograms were quality-checked, and forward and reverse reads were compiled using Geneious Prime^®^ v. 2024.0.3 (Biomatters Ltd., Auckland, New Zealand). For species identification, consensus sequences were subjected to an NCBI BLAST search (http://www.ncbi.nlm.nih.gov/BLAST/) and submitted to the GenBank database.

### 2.4 Pathogenicity test

Two-year-old *C. betulus* saplings were grown from seeds in a 3:1 (v:v) mixture of peat (Pešterski treset, Serbia) and perlite (Agroperlite, Termika, Serbia). One obtained isolate (UBFF161; GenBank accession: ITS– PP843589) was grown on Malt Extract Agar (MEA) in 90 mm plastic Petri dishes (20 ml per dish) and incubated at 20 °C in the dark for two weeks.

Healthy *C. betulus* branches were used to prepare disc-shaped wood and bark fragments (approx. 2–3 mm thick and 6 mm in diameter) using a 6-mm cork borer. Following autoclaving at 120 °C for 15 minutes, the cooled fragments were placed wood-side-down onto the surface of two-week-old cultures. These were then incubated at 20 °C in the dark for four weeks to allow for colonization (Milenković et al., 2025).

At approximately 15 cm above the collar zone of the two-year-old saplings, the bark was surface-sterilized with cotton soaked in 70% ethanol. Ten plants were wounded using a sterile 6-mm cork borer and inoculated with wood and bark fragments colonized by fungal mycelium. For the control group, ten plants were mock-inoculated with sterile wood fragments. Additionally, ten plants were inoculated with wood fragments colonized by *C. carpinicola* mycelium for comparison. The average diameter of the saplings at the collar zone was 4.6 ± 0.25 mm, and the average height of the plants was 367.9 ± 24.27 mm. All inoculation sites were sealed with Parafilm^®^ M and aluminum foil, and the plants were incubated in a glasshouse at approximately 25 °C. A careful visual inspection of all plants was performed weekly. The experiment was concluded six weeks post-inoculation, once at least 50% of the plants exhibited dieback symptoms. The total number of dead plants, plants with girdled necroses, and those with non-girdling necroses was recorded. Re-isolations were performed from the necrotic zones and the margins of the injured woody tissues for all experimental plants. Necrosis lengths were measured with a ruler, and widths were determined using a measuring tape; the total necrotic area was then calculated using the formula for an ellipse. Finally, the mean (x̄) and standard error (± SE) for necrosis length, width, and area were calculated.

### 2.5 Statistical analysis

Data about necrosis length, width, and area were tested for normality and homogeneity by the Shapiro-Wilk’s and Levene’s tests, respectively. As our data didn’t have a normal distribution, and variances were highly nonhomogeneous, one-way PERMANOVA was applied, followed by pairwise comparisons, to test the significance of differences between specific experimental groups (PAST software, version 4.03). Figures were plotted in OriginPro software (Version 2026. OriginLab Corporation, Northampton, MA, USA.).

## 3 Results

### 3.1 Isolation and identification

On the surface of the dead bark within the cankers, thin, dark stromata containing numerous ascomata were formed (**Figures 1C–E**, **2A**). Perithecia were arranged in a single, dense layer with furrowed apexes (**Figure 2B**). Cross-sections revealed immersed, flask-like, dark brown to black perithecia that were ovoid to subglobose in shape with well-developed necks (**Figure 2C**). Asci were club-shaped with long stalks, each containing eight ascospores and averaging 58.5 ± 1.09 × 6.9 ± 0.11 µm (N = 43). Ascospores were uniseriate to biseriate, brown to dark brown, unicellular, and ellipsoid to oval, averaging 7.3 ± 0.15 × 2.8 ± 0.05 µm (N = 54). At the edges of cankers and lesions on the hornbeam bark, characteristic *Cytospora*-like conidiomata were recorded, featuring well-developed, dark orange or red tendrils (**Figures 1G, H**; **Figure 2D**). These tendrils contained numerous unicellular, hyaline, and allantoid (peanut-shaped) conidia (**Figure 2E**) with dimensions averaging 9.5 ± 0.12 × 1.2 ± 0.02 µm, and a range of 7.6−11.7 × 0.95−1.5 µm (n=50). These were formed on straight conidiophores.

In the isolation tests, all 20 collected bark samples were positive on MEA, and 40 pure isolates were subcultured for further maintenance in the culture collection. Colonies were white, dense, cottony, and mainly appressed to the medium across all four tested media (PDA, MEA, V8-A, and CA) (**Figures 2C–F**). The underside of the colonies was white initially and gradually turned light green and then dark green with age, particularly on PDA. The optimum temperature for the growth of the four tested *E. decipiens* isolates was 27.5 °C, with average growth rates of 6.0 ± 0.18 mm/day and 7.1 ± 0.84 mm/day on MEA and PDA, respectively. The maximum temperature for growth was 35 °C; at 37.5 °C, the isolates stopped growing after three days but remained viable, resuming growth when transferred to 20 °C.

The DNA sequence analyses confirmed the morphological identification and presence of *E. decipiens* in the necrotic tissue of *C. betulus*. In a BLAST search, both isolates sequenced belonged to *Anthostoma*/*Eutypella decipiens*. The sequences were submitted to GenBank under the following accession numbers for isolates UBFF161 and UBFF234, respectively: *β-tubulin* (PX906961, PX906962), *cmdA* (PX906963, PX906964), *tef1-α* (PX906965, PX906966), and *ITS* (PP843589, PP467720).

### 3.2 Pathogenicity test

**Figure 3:**
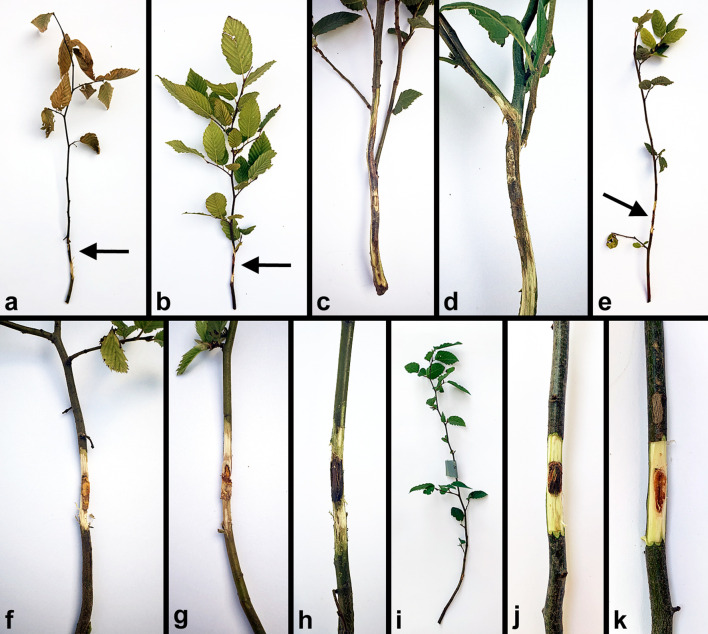
Representative symptoms on two-year-old *Carpinus betulus* plants six weeks after inoculation with *Eutypella decipiens* and *Cryphonectria carpinicola*. **(A)** a dying plant inoculated with *E. decipiens* with exposed necrosis (arrow); **(B)** a living plant inoculated with *E. decipiens* with exposed necrosis (arrow); **(C, D)** extensive necroses caused by *E. decipiens*; **(E)** a declining plant inoculated with *C. carpinicola* with exposed necrosis (arrow); **(F–H)** necroses caused by *C. carpinicola*; **(I–K)** mock-inoculated asymptomatic control plants.

During the incubation period, the first symptoms appeared as early as two weeks post-inoculation (p.i.) in the *E. decipiens* treatment, with two plants showing signs of necrosis near the inoculation sites. Three weeks p.i., the first two plants showed signs of wilting, and prominent necrosis was recorded on three additional plants. By four weeks p.i., a total of five plants were wilting in the *E*. *decipiens* treatment, whereas one plant showed signs of wilting in the *C. carpinicola* treatment. By five weeks p.i., all plants in the *E. decipiens* group and six plants in the *C. carpinicola* group exhibited visible necrosis. At the end of the trial, six weeks p.i., five plants had declined (**Figure 3A**) and one showed dieback in the *E. decipiens* treatment, while a single plant declined in the *C. carpinicola* treatment. Additionally, all plants in both treatments exhibited visible necroses (**Figures 3A–H**). Average necrosis lengths, widths and area in the *E. decipiens* treatment were 100 ± 23.01 mm, 9.3 ± 0.75 mm, and 785.4 ± 244.64 mm^2^, respectively. In *C. carpinicola* treatment necrosis lengths, widths and areas averaged 28.3 ± 14.59 mm, 7.4 ± 1.38 mm, and 210.6 ± 131.76 mm^2^. Control plants showed no symptoms (**Figure 3I**) and wounded points were closed with callus tissues (**Figures 3J, K**). Average necrosis lengths, widths and areas were 9.8 ± 0.67 mm, 4.3 ± 0.63 mm, and 34.3 ± 6.36 mm2, respectively.

**Figure 4:**
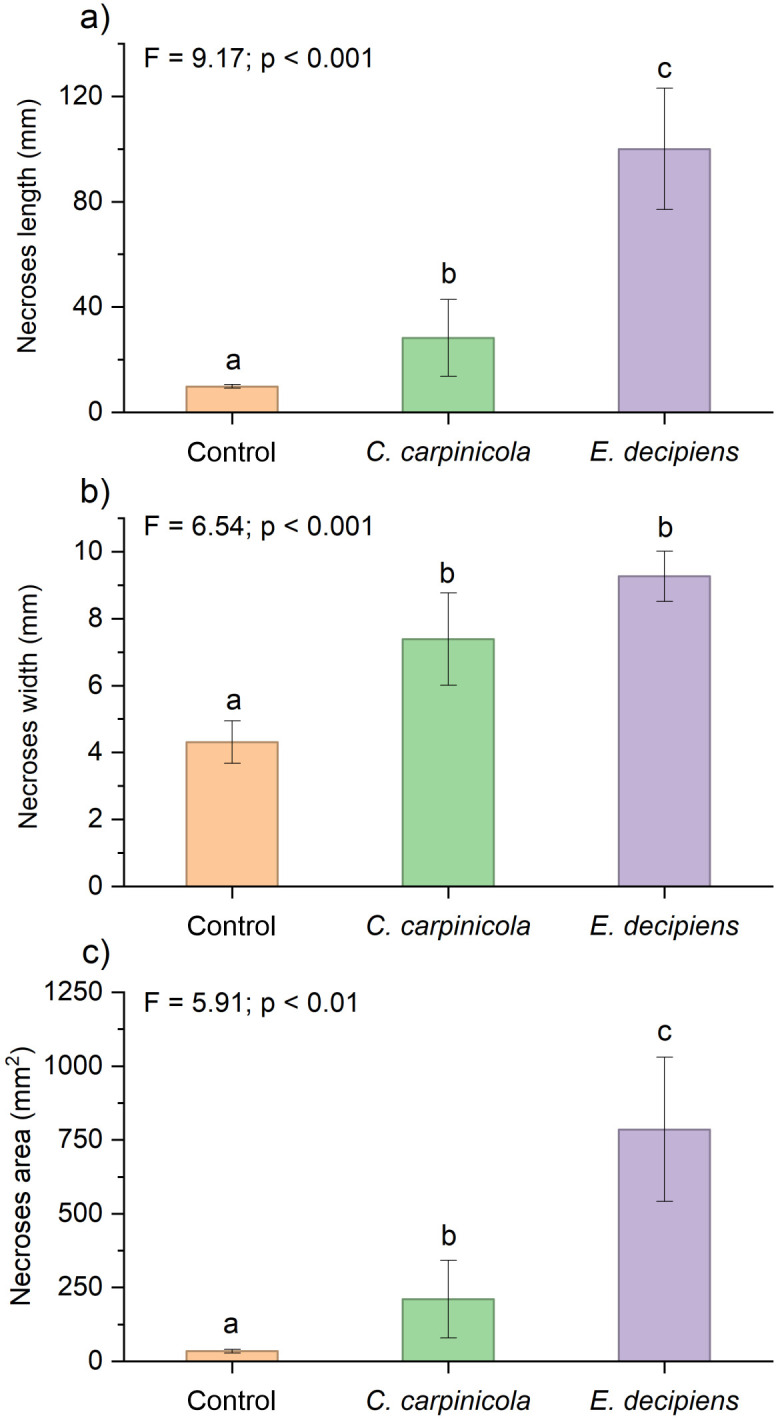
Results of the pathogenicity test on the *Carpinus betulus* seedlings: **(A)** necroses length; **(B)** necroses width; **(C)** necroses area. Different lowercase letters indicate significant differences between treatments.

A significant difference was observed between inoculated and control seedlings in necrosis length (F = 9.17, p < 0.001), necrosis width (F = 6.54, p < 0.001), and necrosis area (F = 5.91, p < 0.01) (**Figure 4**). *Eutypella decipiens* produced necroses with a significantly larger necrosis area (p < 0.05) (**Figure 4C**), that extended more rapidly longitudinally (p < 0.05) compared to *C. carpinicola* (**Figure 4A**). However, no significant difference was found in necrosis width between the two pathogens (p = 0.247) (**Figure 4B**).

## 4 Discussion

Our study identified the fungus *Eutypella decipiens* as the causal agent of the extensive spindle-shaped bark cankers of *C. betulus* trees observed in the urban area. In pathogenicity tests, the fungus caused extensive necrosis and mortality in young *C. betulus* saplings confirming Koch’s Postulates. The recorded necroses were 10.2 times longer and 22.9 times larger in area than those of the mock-inoculated control plants. Furthermore, smaller bark lesions, 2.9 times longer and 6.1 times larger in area compared to the control, were recorded in the *C. carpinicola* treatment. These findings were expected, as similar results were reported by Saracchi et al. (2015), and *E. decipiens* was identified being involved in *C. betulus* decline (Rocchi et al., 2010). However, given the observed mortality and necrosis in our study, the infection potential of *C. carpinicola* should not be overlooked. According to Saracchi et al. (2015), since its description (Saccardo, 1882) *E. decipiens* has a long history of occurrence on *C. betulus* in many regions of Europe and North America. Among neighboring countries, it was recorded in Hungary during the mid-20th century on dead bark of hornbeam and on dead wood of sycamore (*Acer pseudoplatanus* L.) (Tóth, 1967), as well as on other decaying wood (Tóth, 1994). Recently, its presence on *C. betulus* was recorded in several regions across Europe (Rocchi et al., 2010; Karadžić, 2011; Cech, 2019; Muser and Burgdorf, 2022) and in Iran (Mirabolfathy et al., 2018). According to Zhu et al. (2021), the distribution of this fungus includes Asia, Europe, North America, and South America. In addition to hornbeam, *E. decipiens* has been documented contributing to the decline of hazel (*Corylus avellana* L.) (Linaldeddu et al., 2016; Martino et al., 2025) and grapevine (Luque et al., 2012). Furthermore, other ecologically and taxonomically similar species have shown susceptibility to *A. decipiens* infection, including black alder (*Alnus glutinosa* (L.) Gaertn.), common birch (*Betula pendula* Roth), sweet chestnut (*Castanea sativa* Mill.), beech (*F. sylvatica*), and hop hornbeam (*Ostrya carpinifolia* Scop.) (Saracchi et al., 2015). Significantly, most of these species are frequently planted alongside *C. betulus* in horticultural settings in Serbia as ornamental, park, or street trees, presenting a high risk for future disease transmission and widespread infection. In addition to *C. betulus*, *Acer pseudoplatanus* L. trees were planted in the studied alley but decline symptoms and bark cankers were registered exclusively on *C. betulus*.

It is also worth noting that recent taxonomic changes and the latest review of the Diatrypaceae family (Dissanayake et al., 2025) placed the genus *Anthostoma* into *Eutypella*, proposing the new combination *Eutypella decipiens* (Nitschke) Dissan., Hyde & Liu. However, according to the MycoBank and Westerdijk Institute databases, *E. decipiens* is currently treated as a *nomen invalidum* or an obligate synonym, respectively. In these databases, *Anthostoma decipiens* remains the accepted scientific name for this fungus.

*Carpinus betulus* is widely regarded as a durable and adaptable tree, noted for its resistance to both pruning and drought, which has made it a staple species in urban horticulture. Beyond its horticultural value, the ecological significance of *C. betulus* in natural stands is immense, as it serves as a foundational component of forest structure and nutrient cycling. While climate modeling by Koch et al. (2022) suggests that the species may not experience significant shifts relative to its current range, other research highlights its potential as a climate-resilient alternative. For instance, Schmucker et al. (2023) proposed *C. betulus* as a replacement for *F. sylvatica* on wetter sites, as the latter is a mesophilic species highly vulnerable to ongoing climatic changes. However, in the lowland floodplain forests of Serbia, *C. betulus* acts as a hygrophilic associate of *Q. robur*, whose rapid growth and competition for light and space can significantly hinder oak regeneration (Bobinac, 2008; Bobinac et al., 2019). Despite its perceived resilience, more recent projections by Varol et al. (2022) using Maximum Entropy (MaxEnt) modeling under RCP 4.5 and 8.5 scenarios suggest that global climate change will substantially alter the distribution of both *C. betulus* and its sister species *C. orientalis* by 2100. Their findings indicate population losses exceeding 25% below 1600 m altitude for *C. betulus* and 30% below 1000 m for *C. orientalis*. While Beloiu et al. (2020) demonstrated both high susceptibility and a high recovery rate in *C. betulus* saplings following drought stress, Stojnić et al. (2016) found that mature trees respond negatively, often leading to physiological decline. This is corroborated by Scharnweber et al. (2020), who recorded a significant growth decline under drought stress, with the species maintaining only 29.4% of its reference growth rates. Increased sensitivity of plant species to drought stress can lead to a higher incidence of plant diseases; indeed, a positive correlation between drought and disease has been previously documented (Desprez-Loustau et al., 2006). Consequently, in *C. betulus* stands affected by drought, further outbreaks of *E. decipiens* infections are to be expected, especially as the spread of this fungus has been recently recorded (Muser and Burgdorf, 2022; Remmele et al., 2025). These findings should be integrated into future management and silvicultural plans, which should prioritize maintaining dense stands and avoiding sudden canopy openings. However, the concepts argued by Manion (2003), that forests require a baseline level of mortality to remain sustainable should also be considered when managing *C. betulus* stands. In a natural forest, *E. decipiens* might be viewed as a regulator. If a hornbeam stand is too dense, this fungus may help maintain the ‘baseline mortality’ required for the forest’s structural health.

The observed spread of *E. decipiens* in urban areas may also be attributed to climate change, which causes physiological weakening in hornbeam trees (Rocchi et al., 2010), and mechanical injuries sustained during tree maintenance. Also, Karadžić (2011) characterizes *E. decipiens* as an opportunistic pathogen of weakened trees. However, the exact mechanisms of its transmission and infection remain insufficiently understood (Muser and Burgdorf, 2022). Our study indicates that sunburn damage directly contributed to infection by creating suitable entry points for pathogen propagules. *Carpinus betulus*, like beech and other species with smooth thin bark, is highly sensitive to solar radiation, particularly their south- and southwest- exposed sides of the stem (Levitt, 1980). This was precisely the case in our study; significant damage appeared on hornbeam trees, facilitating pathogen penetration. Bark necroses occurred exclusively on the south and southwest-facing sides of individual trees at heights of approximately 1.5 meters, where the incidence of the sun’s rays was greatest. Excessive heating of living cambium cells beneath thin bark, caused by direct solar radiation, leads to sunburn-related heat injury (Levitt, 1980) and eventual cell death (van Doorn et al., 2011). Following necrosis and cracking of the bark, the damaged tissues atrophied and *E. decipiens* infection occurred. As the pathogen developed within the cambial tissue, characteristic elongated, spindle-shaped cankers appeared. Similar symptoms were observed in our pathogenicity tests (**Figure 3**), supporting this theory.

Another negative impact on trees in both urban and naturally growing forest areas is the colonization of cracks and infection sites by decay and rot fungi. This often follows primary infections by typical bark pathogens, such as *E. decipiens* on *C. betulus* or *Phytophthora* species on *F. sylvatica* (Corcobado et al., 2020). Specifically, during our study of several affected trees, fruiting bodies of the cosmopolitan decay fungus *Schizophyllum commune* Fr., the cause of white sapwood rot, were recorded on the margins of cankers (**Supplementary Figure 2**). Therefore, a general recommendation for urban greenery is that thin-barked species, such as hornbeams, planted in south to south-west expositions should be protected from solar radiation. This can be achieved by painting the stems or installing protective materials (woven reeds, slats, or plastic/textile screens) to shade the trunks up to the level of the first branches. By preventing direct solar radiation on the bark, the risk of sunscald and subsequent infection by pathogenic fungi will be reduced. Furthermore, all trees must be thoroughly inspected; areas affected by sunscald should be disinfected and cankers sealed with appropriate protective substances. Continuous monitoring and shading of these trees are mandatory. In cases of advanced cankers and damage, such as those recorded in our study, tree replacement should be considered due to the high probability of colonization by wood-decay fungi.

## Data Availability

The datasets presented in this study can be found in online repositories. The names of the repository/repositories and accession number(s) can be found below: https://www.ncbi.nlm.nih.gov/genbank/, PX906961, PX906962, PX906963, PX906964, PX906965, PX906966, PP843589, PP467720. Data are available on reasonable request.

## Supplementary material

Supplementary Figure 1 Orientation of the Knjaz Miloš street in Aranđelovac, central Serbia, determined using Google Earth Pro (v. 7.3.6, Google Inc., Mountain View, CA).

Supplementary Figure 2 *Carpinus betulus* tree infected with *Eutypella decipiens*, and with visible fruiting bodies of *Schizophyllum commune*: **(A)** large longitudinal canker with *E. decipiens* tendrils (yellow arrows), and with fruiting bodies of *S. commune* (white arrows); **(B)** fruiting bodies of *S. commune*.

Supplementary Table 1 PCR conditions and primers used for amplification and sequencing of *Eutypella decipiens*.
