# Supplementary material for: Sunburn-induced bark damage facilitates Eutypella decipiens infection of Carpinus betulus in Serbian urban landscapes
Source: Front Plant Sci. 2026 May 11;17:1828539. doi: 10.3389/fpls.2026.1828539 (PMC13199238; doi:10.3389/fpls.2026.1828539)
Supplement: Supplementary Figure 1 — Orientation of the Knjaz Miloš street in Aranđelovac, central Serbia, determined using Google Earth Pro (v. 7.3.6, Google Inc., Mountain View, CA). [file Table2.docx]

Supplementary Material


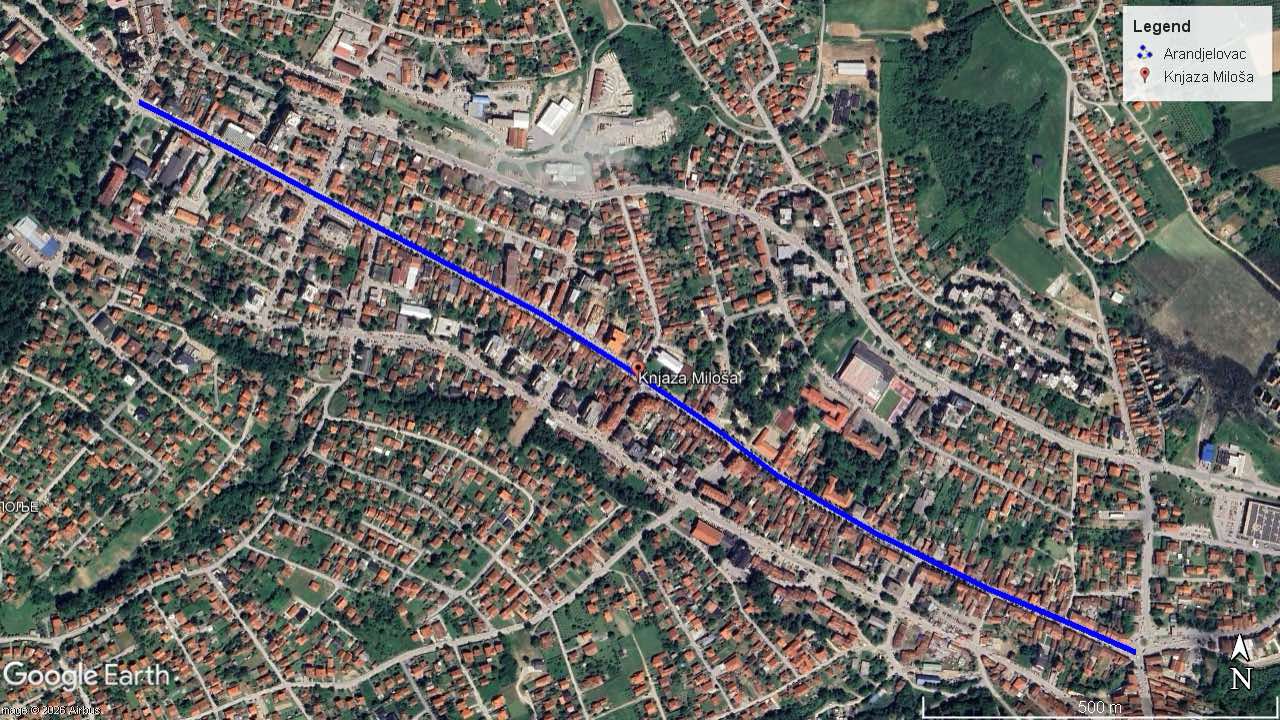


**Supplementary Figure 1.** Orientation of the Knjaz Miloš street in Aranđelovac, central Serbia, determined using Google Earth Pro (v. 7.3.6, Google Inc., Mountain View, CA).
