# Supplementary material for: Sunburn-induced bark damage facilitates Eutypella decipiens infection of Carpinus betulus in Serbian urban landscapes
Source: Front Plant Sci. 2026 May 11;17:1828539. doi: 10.3389/fpls.2026.1828539 (PMC13199238; doi:10.3389/fpls.2026.1828539)
Supplement: Supplementary Figure 2 — Carpinus betulus tree infected with Eutypella decipiens, and with visible fruiting bodies of Schizophyllum commune: (A) large longitudinal canker with E. decipiens tendrils (yellow arrows), and with fruiting bodies of S. commune (white arrows); (B) fruiting bodies of S. commune. [file Table3.docx]

Supplementary Material


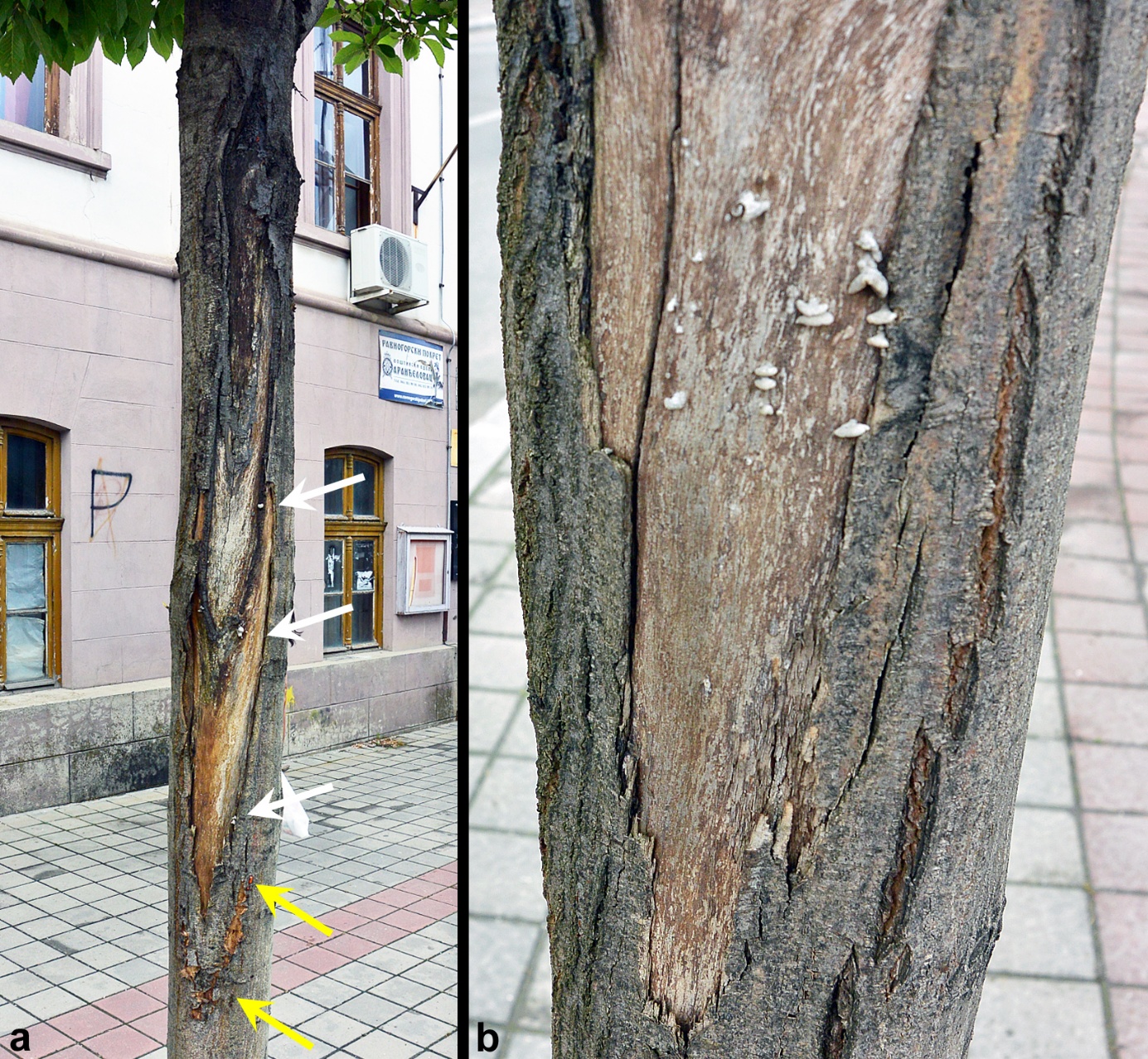


**Supplementary Figure 2.** *Carpinus betulus* tree infected with *Eutypella decipiens*, and with visible fruiting bodies of *Schizophyllum commune*: (a) large longitudinal canker with *E. decipiens* tendrils (yellow arrows), and with fruiting bodies of *S. commune* (white arrows); (b) fruiting bodies of *S. commune*.
