# Supplementary material for: Sunburn-induced bark damage facilitates Eutypella decipiens infection of Carpinus betulus in Serbian urban landscapes
Source: Front Plant Sci. 2026 May 11;17:1828539. doi: 10.3389/fpls.2026.1828539 (PMC13199238; doi:10.3389/fpls.2026.1828539)
Supplement: Supplementary Table 1 — PCR conditions and primers used for amplification and sequencing of Eutypella decipiens. [file Table1.docx]

Supplementary Material

**Table 1.** PCR conditions and primers used for amplification and sequencing of *E. decipiens*.

| **Locus** | **Primer names** | **Primer sequences (5’-3’)** | **Orientation** | **Annealing temperature (°C)** | **Exstension time (s)** | **Reference for primer sequences** |
| --- | --- | --- | --- | --- | --- | --- |
| βtub | Bt1a | TTCCCCCGTCTCCACTTCTTCATG | forward | 65 | 8 | Glass and Donaldson (1995) |
|  | Bt1b | GACGAGATCGTTCATGTTGAACTC | reverse | 65 | 8 |  |
|  | Bt2a | GGTAACCAAATCGGTGCTGCTTTC | forward | 69 | 8 |  |
|  | Bt2b | ACCCTCAGTGTAGTGACCCTTGGC | reverse | 69 | 8 |  |
| cmdA | CL2F | GACAAGGAYGGYGATGGT | forward | 62 | 12 | Duong et al. (2012) |
|  | CL2R | TTCTGCATCATGAGYTGSAC | reverse | 62 | 12 |  |
| *tef*1α | EF1-728F | CATCGAGAAGTTCGAGAAGG | forward | 63 | 12 | Carbone and Kohn (1999) |
|  | EF-2 | GGARGTACCAGTSATCATGTT | reverse | 63 | 12 | O’Donnell et al. (1998) |
| ITS | ITS5 | GGAAGTAAAAGTCGTAACAAGG | forward | 65 | 12 | White et al. (1990). |
|  | ITS4 | TCCTCCGCTTATTGATATGC | reverse | 65 | 12 |  |
